# Supplementary material for: An Observational Prospective Cohort Study of Incidence and Outcome of Streptococcus pneumoniae and Hemophilus influenzae Infections in Adult Solid Organ Transplant Recipients
Source: Microorganisms. 2021 Jun 24;9(7):1371. doi: 10.3390/microorganisms9071371 (PMC8304095; doi:10.3390/microorganisms9071371)
Supplement: Supplementary file 1 [file microorganisms-09-01371-s001.zip › microorganisms-1211647-supplementary.pdf]

Supplementary Table S1: Characteristics of the matched cases and controls who had and did not have S. pneumoniae infection.

**Variable**		**At least one positive culture for *S. pneumoniae* (n=34)**	**Matched SOT recipients with no positive culture for *S. pneumoniae* (n=102)**	**Total (n=136)**	**p-value**
**Age, median (IQR)**		51 (38, 58)	55 (41, 61)	54 (39, 60)	0.24
**Male (n, %)**		22 (65)	66 (65)	88 (65)	1.0
**Transplanted organ (n, %)**	**Heart**	3 (8.8)	8 (7.8)	11 (8.1)	0.94
	**Kidney**	15 (44)	42 (41)	57 (42)	
	**Liver**	10 (29)	36 (35)	46 (34)	
	**Lung**	6 (18)	16 (16)	22 (16)	
**Days of follow up post-transplantation, median (IQR)**		1606 (1089, 1826)	1672 [1078, 1826]	1,657 (1,083, 1,826)	0.99
**Death, yes (n, %)**		4 (12)	12 (12)	16 (12)	1.0

Supplementary Table S2: Characteristics of the matched cases and controls who had and did not have H. influenzae infection

**Variable**		**At least one positive culture for *H. influenzae* (n=44)**	**Matched SOT recipients with no positive culture for *H. influenzae* (n=132)**	**Total (n=176)**	**p-value**
**Age, median (IQR)**		55 (46, 60)	54 (44, 60)	54 (44, 60]	0.61
**Male (n, %)**		26 (59)	72 (55)	98 (56)	0.73
**Transplanted organ (n, %)**	**Heart**	1 (2.3)	4 (3.0)	5 (2.8)	0.93
	**Kidney**	24 (55)	65 (49)	89 (51)	
	**Liver**	10 (23)	35 (27)	45 (26)	
	**Lung**	9 (21)	28 (21)	37 (21)	
**Days of follow up post-transplantation, median (IQR)**		1528 (1100, 1,826)	1826 (1058.2, 1826)	1757 (1086, 1,826)	0.23
**Death, yes (n, %)**		4 (9.1)	16 (12)	20 (11)	0.78
